# Excellent Thermoelectric Properties in monolayer WSe_2_ Nanoribbons due to Ultralow Phonon Thermal Conductivity

**DOI:** 10.1038/srep41418

**Published:** 2017-01-25

**Authors:** Jue Wang, Fang Xie, Xuan-Hao Cao, Si-Cong An, Wu-Xing Zhou, Li-Ming Tang, Ke-Qiu Chen

**Affiliations:** 1 Department of Applied Physics, School of Physics and Electronics, Hunan University, Changsha 410082, China; 2 Hunan Province Higher Education Key Laboratory of Modeling and Monitoring on the Near-Earth Electromagnetic Environments, Changsha University of Science and Technology, Changsha 410004, China.

## Abstract

By using first-principles calculations combined with the nonequilibrium Green’s function method and phonon Boltzmann transport equation, we systematically investigate the influence of chirality, temperature and size on the thermoelectric properties of monolayer WSe_2_ nanoribbons. The results show that the armchair WSe_2_ nanoribbons have much higher ZT values than zigzag WSe_2_ nanoribbons. The ZT values of armchair WSe_2_ nanoribbons can reach 1.4 at room temperature, which is about seven times greater than that of zigzag WSe_2_ nanoribbons. We also find that the ZT values of WSe_2_ nanoribbons increase first and then decrease with the increase of temperature, and reach a maximum value of 2.14 at temperature of 500 K. It is because the total thermal conductance reaches the minimum value at 500 K. Moreover, the impact of width on the thermoelectric properties in WSe_2_ nanoribbons is not obvious, the overall trend of ZT value decreases lightly with the increasing temperature. This trend of ZT value originates from the almost constant power factor and growing phonon thermal conductance.

Thermoelectric material, which can directly convert waste heat into electricity and vice versa, have attracted major attentions in recent years due to increasing world energy consumption and decreasing fuel supply^1^,^2^. Current applications of thermoelectric energy conversion technology, however, are severely hindered by the limited thermoelectric conversion efficiency, which is quantified by a dimensionless thermoelectric figure of merit, defined as *ZT* = *S*^2^ ⋅ *G* ⋅ *T*/*κ*, where *S, G, T* and *κ* are Seebeck coefficient, electronic conductance, absolute temperature and thermal conductance (include both the phononic and electronic contributions), respectively. According to the formula, in order to obtain a high *ZT*, high electronic conductance *G*, high Seebeck coefficient *S*, as well as low thermal conductance *κ* are required. Nevertheless, it is very difficult to modify one quantity independently and keep the other quantities unaffected^3^,^4^. For instance, the materials with the large electrical conductivity usually have small Seebeck coefficient and large electrical thermal conductivity because of the Wiedemann-Franz relation. Until the 1990 s, Hicks *et al*. theoretically predicated that nanostructuring of materials may improve the thermoelectric properties owing to reduced lattice thermal conductivity caused by phonon-boundary scattering and increased power factor caused by quantum confinement^5^. From then on many kinds of nanostructures materials, such as superlattices^6^,^7^, nanoribbons^8^,^9^,^10^, nanowires^11^,^12^,^13^,^14^,^15^, etc, are investigated to seek the high quality thermoelectric materials.

Recent years, transition metal dichalcogenides (TMDCs), as a family of two-dimensional materials, have attracted a lot of attentions due to their potential applications in field-effect transistors, photoelectric devices, thermoelectric devices and so on^16^,^17^,^18^,^19^,^20^,^21^,^22^,^23^. MoS_**2**_ monolayers, as a typical semiconducting TMDCs, is regarded as a great candidate for thermoelectric applications recently due to the low thermal conductivity and large Seebeck coefficient^24^,^25^,^26^,^27^,^28^,^29^. Nevertheless, WSe_**2**_ monolayers, have a MoS_**2**_-like two-dimensional structure, has much lower thermal conductivity than MoS_**2**_ monolayers^30^,^31^. Chiritescu *et al*. measured the cross-plane thermal conductivity of disordered WSe_**2**_ thin films can be as low as 0.05 W/mK^32^, which is the lowest lattice thermal conductivity ever reported for a dense solid. Soon after, Shi *et al*. measured the in-plane lattice thermal conductivity of disordered WSe_**2**_ thin films^33^, and found that the in-plane lattice thermal conductivity of the disordered layered WSe_**2**_ thin films is about six times lower than that of compacted single-crystal platelets. Very recently, Zhou *et al*. systematically investigate the lattice thermal conductivity of WSe_**2**_ monolayers by using first-principles calculations combined with the phonon Boltzmann transport equation^34^, and found the WSe_**2**_ monolayers have an ultralow thermal conductivity due to the ultralow debye frequency and heavy atom mass. In addition, compared with MoS_**2**_ monolayers, WSe_**2**_ monolayers have a much narrower band gap^35^, which can offer a good balance of the electronic conductivity and Seebeck coefficient. Therefore, these researches suggest that the monolayer WSe_**2**_ has great potential in thermoelectric applications.

However, the systematical research on thermoelectric properties of monolayer WSe_**2**_ is still in its infancy. In the present work, we investigate the thermoelectric properties of monolayer WSe_**2**_ systematically by using first-principles calculations combined with the phonon Boltzmann transport equation (PBTE) and nonequilibrium Green’s function (NEGF) method. Firstly, we study the chirality effect of monolayer WSe_**2**_ nanoribbons on thermoelectric properties, and find that the armchair WSe_**2**_ nanoribbons have much higher ZT value than zigzag WSe_**2**_ nanoribbons. The ZT value of armchair WSe_**2**_ nanoribbons can reach 1.4 at room temperature, which is about seven times greater than that of zigzag WSe_**2**_ nanoribbons. Moreover, we also discuss the influence of temperature and width on thermoelectric properties in WSe_**2**_ nanoribbons, and find that the ZT value increase first and then decrease with the increase of temperature, and reach a maximum value of 2.14 at temperature of 500 K. However, the impact of width on the thermoelectric properties in WSe_**2**_ nanoribbons is not serious, the overall trend of ZT value decreases lightly with the increasing temperature. These trends of ZT value originate from the competition between power factor and thermal conductivity.

## Results

**Figure 1:**
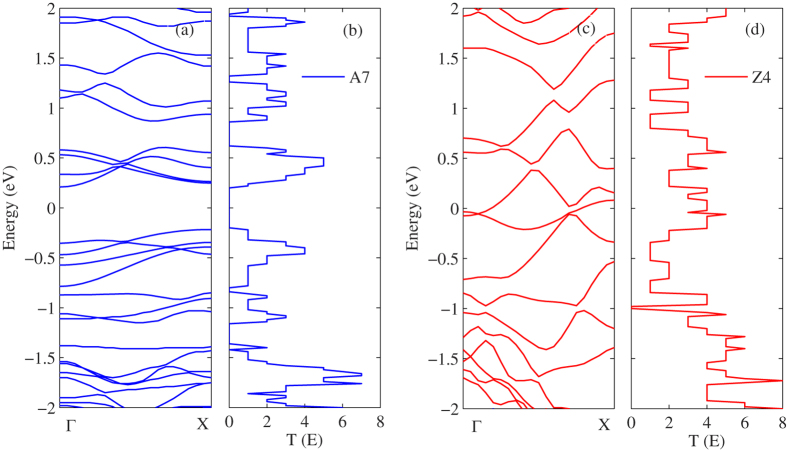
Energy band structure and electron transmission function for (**a**) A7 and (**b**) Z4.

Similar to graphene nanoribbons, WSe_**2**_ nanoribbons also can be zigzag-edged or armchair-edged denoted as A*n* and Z*n*, respectively, where *n* denotes the width of WSe_**2**_ nanoribbons. Firstly, we research the chirality effect of monolayer WSe_**2**_ nanoribbons on thermoelectric properties. For comparison purposes, we chose the A7 and Z4 as the research objects because they have the same width of 1 nm. To understand the thermoelectric transport properties, we first research the electronic transport properties of WSe_**2**_ nanoribbons. Figure 1(a)–(d) show the energy band structure and the electronic transmission function of A7 and Z4, respectively. It is clearly shown that the transmission function of perfect WSe_**2**_ nanoribbons display clear stepwise structure, which indicates that the transport is ballistic, and the electrons from the lead pass through the center region without any scattering. The quantized transmission can also be obtained by counting the numbers of energy bands at any given energy^12^. In addition, it is clearly shown that the A7 is semiconducting, and the Z4 is metallic from Fig. 1(a) and (c). It is interesting that the A7 exhibits a zero transmission window around the Fermi level. It implies that the A7 will have a larger power factor, it is because the Seebeck coefficient is relatively large near the edge of the zero transmission windows.

**Figure 2:**
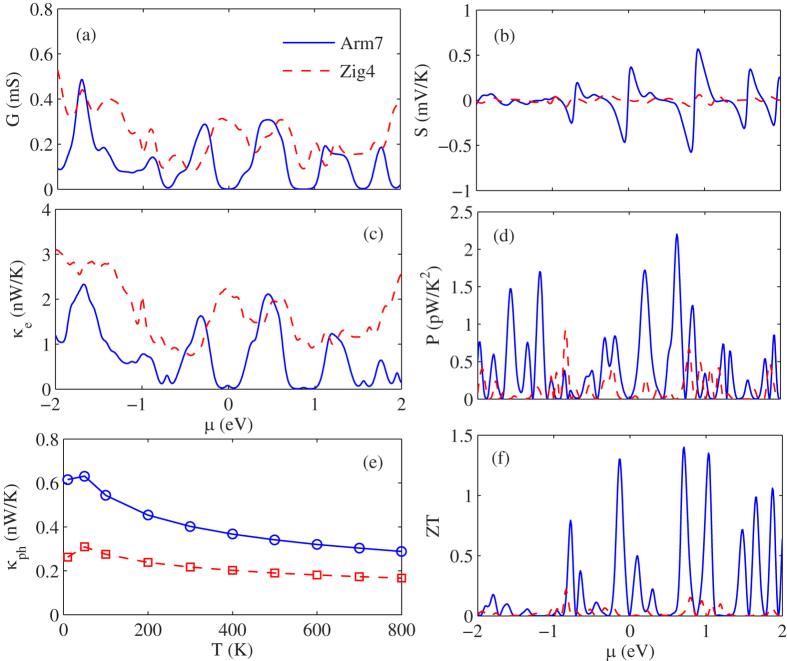
(**a**) Electrical conductance *G*, (**b**) Seebeck coefficient *S*, (**c**) electron thermal conductance *κ*_*e*_, (**d**) power factor *P*, and (**f**) ZT of A7 and Z4 with different chemical potentials at room temperature. (**e**) Phonon thermal conductance *κ*_*ph*_ of A7 and Z4 at different temperatures.

Based on the electronic transmission function, we calculate the electronic conductance *G*, the Seebeck coefficient *S*, the power factor *S*^2^*G* and electronic thermal conductance *κ*_*e*_ of A7 and Z4 with different chemical potential at room temperature, as shown in Fig. 2(a)–(d), respectively. In panel (a), we can clearly see that the electronic conductance of Z4 is larger than that of A7 due to the metallic property. In contrast, the Seebeck coefficient is diametrically opposed to electronic conductance, the A7 have a relatively large Seebeck coefficient, as shown in panel (b). This is because the increase of the electrical conductance will decrease the Seebeck coefficient duo to the usual interdependence of the transport parameters^2^. Therefore, the power factor *S*^2^*G* is depending on the competition between electrical conductance and Seebeck coefficient. In panel (c), we can find that the A7 has a larger power factor than Z4. This result accords closely with our predictions in the part of energy band structure analysis. Panel (d) shows the electronic thermal conductance of the A7 and Z4 at 300 K. As we know, the most important contribution of the thermal conductance comes from phonons in the semiconductor materials and insulation materials, the electronic thermal conductance can be negligible. However, in metallic materials, the electrons have also important contributions to the total thermal conductance. Here, the Z4 has a much large electron conductance than A7 is due to its metallic property. In addition, it is worth noting that the electrons thermal conductance has a similar trend with electronic conductance as shown in Fig. 2(a) and (d), it is because the charge carriers are also heat carriers.

To evaluate the ZT value explicitly, we calculated the temperature dependence of phononic thermal conductance of the A7 and Z4 as shown in Fig. 2(e). We can see that the phononic thermal conductance of A7 and Z4 increase first and then decrease with the increase of temperature. This phenomenon can be understood from the phonon scattering mechanisms. At low temperatures, the Umklapp phonon-phonon scattering is very weak, and a growing number of phonons are excited to participate in thermal transport with the increase of temperature, leading to the phononic thermal conductance increases significantly. However, at high temperature, the Umklapp phonon-phonon scattering is dominant, so the thermal conductance decreases significantly with the increase of temperature. In addition, from Fig. 2(e), we also find that thermal conductance of A7 is significantly higher than that of Z4, the thermal conductance value of A7 is approximately twice than that of Z4. This is because the phonon group velocity along the heat transport direction of A7 is significantly greater than that of Z4^34^. Although the A7 has a higher thermal conductivity than Z4, the ZT of A7 is significantly higher than that of Z4, as shown in Fig. 2(f), the ZT value of A7 can reach 1.4 at room temperature, which is about seven times greater than that of Z4. It is because that the A7 has much higher power factor than Z4 as shown in Fig. 2(c). In addition, it is worth noting that the ZT is heavily depending on the electron chemical potential, we can control the electron chemical potential by chemical doping or electrical gating to get the optimal ZT value^36^. In short, the ZT value is depends on the competition between thermal conductance and power factor.

**Figure 3:**
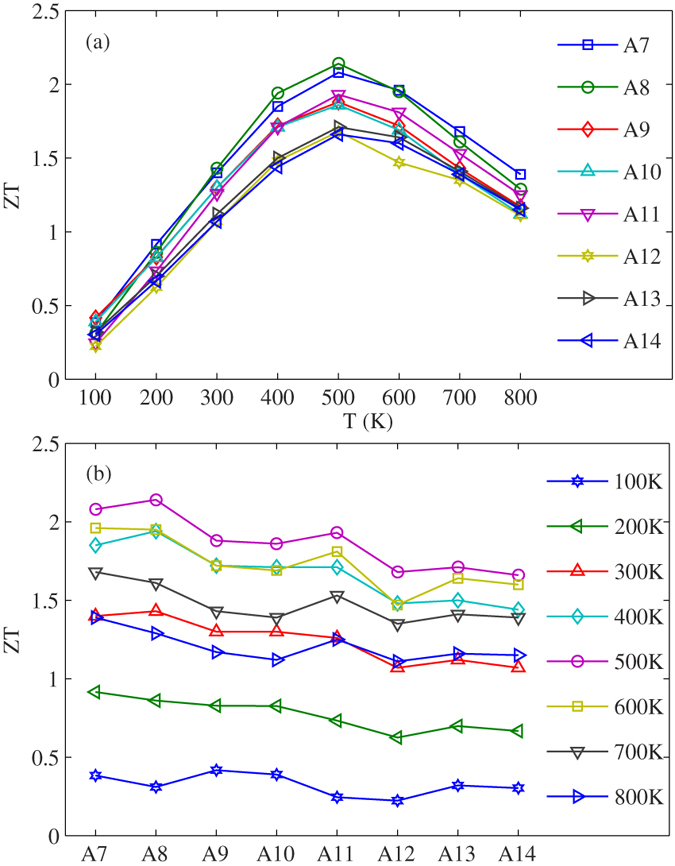
(**a**) The temperature dependence of ZT in monolayer WSe_2_ with different widths. (**b**) The width dependence of ZT in monolayer WSe_2_ under different temperatures.

In order to further improve the thermoelectric performance of WSe_**2**_ nanoribbons, we study the influences of temperature and size on the thermoelectric properties of armchair WSe_**2**_ nanoribbons. We plot the ZT as a function of the temperature and the width of WSe_**2**_ nanoribbons in Fig. 3(a) and (b), respectively. Obviously, the ZT of WSe_**2**_ nanoribbons increases first with the increasing of temperature, and then decreases, as shown in Fig. 3(a). The ZT value can reach a maximum value of 2.14 at temperature of 500 K. And previous experimental result shows that the structure of WSe_2_ is still stable when the temperature is lower than 900K^37^. In addition, from Fig. 3(b) we can find that the influence of width on the thermoelectric properties in WSe_**2**_ nanoribbons is not serious, the overall trend of ZT value decreases lightly with the increasing temperature.

**Figure 4:**
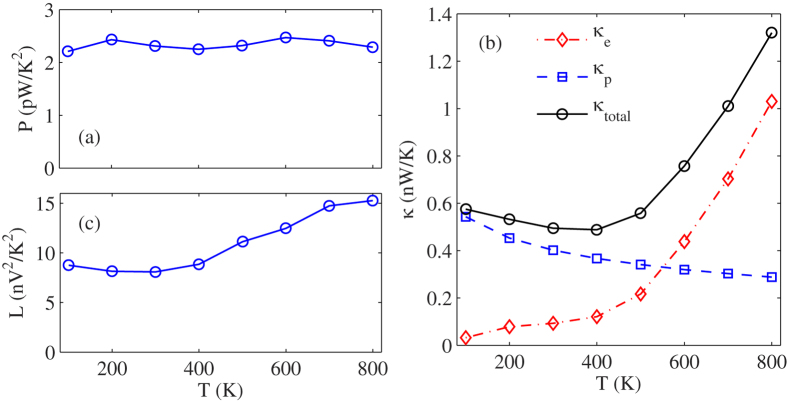
The temperature dependence of (**a**) power factor *P*, (**b**) electron thermal conductance *κ*_*e*_, Phonon thermal conductance *κ*_*ph*_ and total thermal conductance, (**c**) Lorenz number of A7.

To explain the influence of temperature on the thermoelectric properties of WSe_**2**_ nanoribbons, we plot the power factor and thermal conductance of WSe_**2**_ nanoribbons as a function of temperature in Fig. 4(a) and (b), respectively. Here, the power factor and electronic thermal conductance correspond to the optimal values to achieve the maximum value of ZT. From Fig. 4(a), we can clearly see that the power factor of WSe_**2**_ nanoribbons almost keeps constant with the increasing of temperature. It is because the influence of temperature on power factor mainly involve the following aspects: On the one hand, the electronic conductance will be increased with the increasing of temperature due to a growing number of electrons can be excited into the conductance band and contribute to the electronic conductance. But on the other hand, the increase of the electrical conductance will decrease the Seebeck coefficient duo to the usual interdependence of the transport parameters. Therefore, the power factor of WSe_**2**_ nanoribbons depends on the competition between electronic conductance and Seebeck coefficient. In addition, the electrons thermal conductance is also increased with the increasing of temperature as shown in Fig. 4(b), because the charge carriers are also heat carriers. But the phononic thermal conductance is decreased with the increasing of temperature due to the enhanced phonon-phonon interaction. As a result the phonons play the dominant role in thermal transport at low temperature, and the electrons play the leading role in thermal transport at high temperature. So the total thermal conductance of WSe_**2**_ nanoribbons decreases with the increasing of temperature, and then increases. This phenomenon results from the competition between electronic thermal conductance and phononic thermal conductance. In short, the influence of temperature on the thermoelectric properties of WSe_**2**_ nanoribbons is depends on the combined effect from electronic conductance, Seebeck coefficient, phononic thermal conductance and electronic thermal conductance. In addition, we also calculate the Lorenz number for the ratio of conductance and thermal conductance of electrons, and find that the Lorenz number is no longer a constant in WSe_2_ nanoribbons, but increases obviously as the temperature rises.

**Figure 5:**
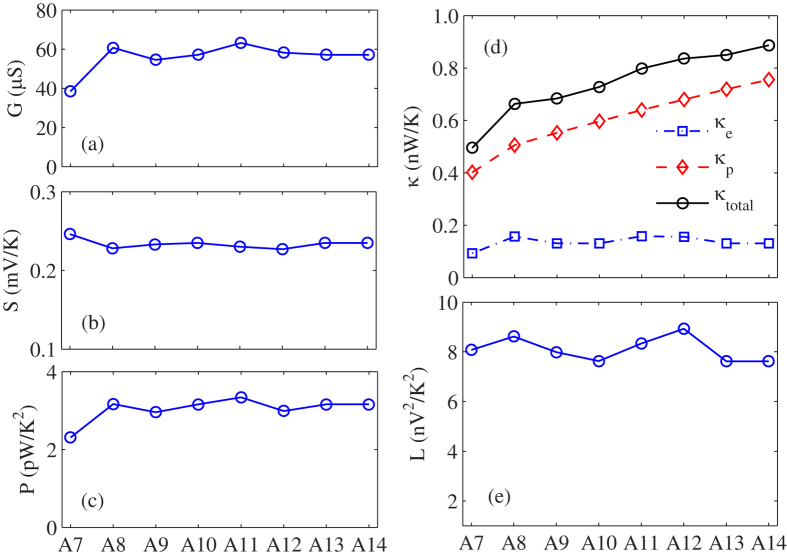
The width dependence of (**a**) Electrical conductance G, (**b**) Seebeck coefficient S, (**c**) power factor *P*, (**d**) electron thermal conductance *κ*_*e*_, Phonon thermal conductance *κ*_*ph*_ and total thermal conductance (**e**) Lorenz number of monolayer WSe_2_ nanoribbons at room temperature.

In order to further understand the size effect of the thermoelectric properties in WSe_**2**_ nanoribbons, we plot the electronic conductance, the Seebeck coefficient, the power factor, thermal conductance and Lorenz number of WSe_**2**_ nanoribbons as a function of width at 300 K in Fig. 5(a–e), respectively. From Fig. 5(a) and (b), we can find that the electronic conductance and the Seebeck coefficient are almost not changed with increasing the nanoribbons width, causing the optimized power factor is almost constant with the increasing of the width as shown in Fig. 5(c). However, the total thermal conductance of WSe_**2**_ nanoribbons increases significantly as the nanoribbons width increases, as shown in Fig. 5(d). We also find that the phononic thermal conductance of WSe_**2**_ nanoribbons dominate the thermal transport due to the semiconducting properties, and increases significantly with the increasing of nanoribbons width. Nevertheless, the electronic thermal conductance is almost constant. So the increase of total thermal conductance in WSe_**2**_ nanoribbons is due to the increase of phononic thermal conductance, which is because the phonon-boundary scattering is weakened with the increasing of nanoribbons width. In addition, we also find that the Lorenz number does not change much as the size increases from Fig. 5(e). In brief, the light decrease of ZT value with the increasing of nanoribbons width originates from the almost constant power factor and growing phononic thermal conductance.

## Discussion

In summary, by using first-principles calculations combined with the nonequilibrium Green’s function method and phonon Boltzmann transport equation, we systematically investigate the influence of chirality, temperature and size on the thermoelectric properties of monolayer WSe_**2**_ nanoribbons. The results show that the armchair WSe_**2**_ nanoribbons have much higher ZT value than zigzag WSe_**2**_ nanoribbons. The ZT value of armchair WSe_**2**_ nanoribbons can reach 1.4 at room temperature, which is about seven times greater than that of zigzag WSe_**2**_ nanoribbons. We also find that the ZT value increase first and then decrease with the increase of temperature, and reach a maximum value of 2.14 at temperature of 500 K. It is because the total thermal conductance reaches the minimum value at 500 K. Moreover, we also find the impact of width on the thermoelectric properties in WSe_**2**_ nanoribbons is not serious, the overall trend of ZT value decreases lightly with the increasing temperature. This trend of ZT value originates from the almost constant power factor and growing phononic thermal conductance.

## Method

We adopt the density functional theory (DFT) as well as non-equilibrium Green’s function method to optimize structure and calculate the electrical transmission, as implemented in Atomistix ToolKit (ATK) software package^38^,^39^. Since the electron-phonon interaction is very weak in WSe_2_ NWs^8^,^40^, in the present work, the electron transport is assumed to travel ballistically. The single-zeta plus polarization (SZP) basis set are employed, and the exchange-correlation potential is described by the local density approximation. The Brillouin zone is sampled with 1 × 1 × 100 Monkhorst-Pack k-mesh, and the cutoff energy is set to 150 Ry. All atomic positions are relaxed until the maximum atomic force becomes smaller than 0.01 eV/A. For electron transport calculation, the retarded Green’s function *G*_*r*_ should be obtained firstly,


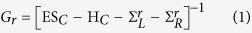


Where E is the electron energy, and S_C_ and H_C_ are the overlap matrix and Hamiltonian matrix of the center scattering region, respectively. 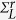 and 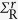 denote the self-energy of the left and right semi-infinite leads, which can be calculated as


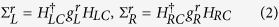


where 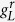 and 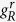 are the retarded surface Green’s function of the left and right lead, respectively. Then, according to the Caroli formula, the electron transmission is calculated as


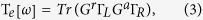


Where *G*^*r*^ = (G^*a*^)^†^ is the retarded Green function of the central scattering region and Γ_*L(R*)_ represents the coupling interaction with the left (right) semi-infinite lead. Thus, the physical quantities involved in the ZT formula can be obtained by, respectively,


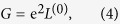



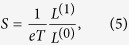



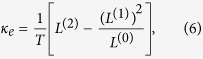


with


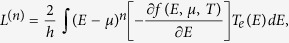


Where *f(E,μ,T*)is the Fermi-Dirac distribution function at the chemical potential *μ* and temperature T.

We calculate the lattice thermal conductance by using first-principles calculations combined with the phonon Boltzmann transport equation with relaxation time approximation. The thermal conductivity in branch *λ* of monolayer WSe_2_ in the longitudinal direction of ribbon is derived as


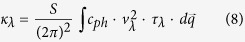


Where λ = TA, LA and ZA^41^. Here we only consider the contribution of three acoustic phonons to the thermal conductivity, because the contribution of optical phonons can be negligible due to the short phonon lifetime and small group velocity^27^. S is the area of the sample, *v*_*λ*_ is the phonon group velocity, which can be calculated as *v*_*λ*_ = *dω*/*dq, ω* is the phonon frequency for branch λ at wave vector q. *c*_*ph*_ is the volumetric specific heat of each mode, which can be written as


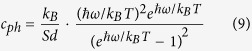


Where *k*_*B*_ is the Boltzmann constant, *d* = 0.648 nm is the effective layer thickness of monolayer WSe_2_, which is assumed to be the interlayer spacing of bulk WSe_2_^42^. 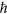 is the reduced Planck constant, and T is the absolute temperature. According to the Matthiessen’s rule, the averaged phonon relaxation time *τ*_*λ*_can be given as


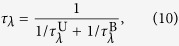


The 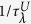, which is the Umklapp phonon-phonon scattering rate, can be written as^43^,^44^


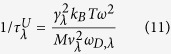


where *γ*_*λ*_ is the Grüneisen parameter, which characterizes the strength of the Umklapp phonon-phonon scattering. M is the mass of a WSe_2_ unit cell, and *ω*_*D,λ*_ is the Debye frequency of branch λ. The scattering rate of phonon-boundary scattering can be given as^41^


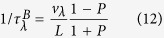


where L is sample size, and P is the specularity parameter, which is defined as a probability of specular scattering at the boundary. These quantities such as phonon dispersion relation, phonon group velocity and Grüneisen parameter are calculated by using the PHONOPY code combined with Vienna ab-initio simulation Package (VASP) based on the density functional theory^45^,^46^,^47^. The project-augmented wave potential and generalized-gradient approximation exchange-correlation functional are adopted in our calculations^48^,^49^. The energy cutoff for the plane-wave expansion is set as 400 eV, and a Monkhorst-Pack k-mesh of 12 × 12 × 1 is used to sample the Brillouin zone, with the energy convergence threshold set as 10^−6^ eV. A 18.41 Å vacuum spacing is used to eliminate the interactions emerging from the employed periodic boundary conditions. The structures are fully relaxed until the maximal forces exerted on the atoms are no larger than 10^−6^ eV/Å.

## Additional Information

**How to cite this article**: Wang, J. *et al*. Excellent Thermoelectric Properties in monolayer WSe_2_ Nanoribbons due to Ultralow Phonon Thermal Conductivity. *Sci. Rep.* **7**, 41418; doi: 10.1038/srep41418 (2017).

**Publisher's note:** Springer Nature remains neutral with regard to jurisdictional claims in published maps and institutional affiliations.
